# A glance at the gut microbiota of five experimental animal species through fecal samples

**DOI:** 10.1038/s41598-020-73985-2

**Published:** 2020-10-06

**Authors:** Zhiguang Xiang, Hua Zhu, Bochao Yang, Hang Fan, Jianguo Guo, Jiangning Liu, Qi Kong, Qingfeng Teng, Haiquan Shang, Lei Su, Chuan Qin

**Affiliations:** 1 Institute of Laboratory Animal Science, Chinese Academy of Medical Sciences, Beijing, China; 2 Beijing Institute of Microbiology and Epidemiology, Beijing, China

**Keywords:** Microbiology, Zoology, Gastroenterology

## Abstract

Experimental animals including the ferret, marmoset, woodchuck, mini pig, and tree shrew have been used in biomedical research. However, their gut microbiota have not been fully investigated. In this study, the gut microbiota of these five experimental animals were analyzed with 16S rRNA sequencing. The phyla Firmicutes, Bacteroidetes, and Fusobacteria were present in the gut microbiota of all the species. Specific phyla were present in different animals: Proteobacteria in the ferret, Tenericutes in the marmoset, and Spirochaetes in the mini pig. *Fusobacterium* and unidentified Clostridiales were the dominant genera in the ferret, whereas *Libanicoccus*, *Lactobacillus*, *Porphyromonas*, and *Peptoclostridium* were specific to marmoset, mini pig, woodchuck, and tree shrew, respectively. A clustering analysis showed that the overall distribution of microbial species in the guts of these species mirrored their mammalian phylogeny, and the microbiota of the marmoset and tree shrew showed the closest *bray_curtis* distances to that of humans. PICRUSt functional prediction separated the woodchuck from the other species, which may reflect its herbivorous diet. In conclusion, both the evolutionary phylogeny and daily diet affect the gut microbiota of these experimental animals, which should not be neglected for their usage in biomedical research.

## Introduction

Research has shown that the gastrointestinal microbiome forms a barrier to pathogens, activates the host immune system, regulates inflammation, and participates in energy metabolism^1^. The gut microbiota have received much research attention because they are associated with human diseases, such as obesity, diabetes, rheumatoid arthritis, and cardiovascular disease^2–5^. Some animals, such as rodents and some nonhuman primates, have been used experimentally to model perturbations in the gut microbiota in order to assess the roles of host microbe interactions and disease associated changes in the gut microbiota composition in diseases^6–9^.

Several other animals, like ferret, marmoset, woodchuck, mini pig, and tree shrew have also been used in biomedical research^10–24^. However, there is limited information on their gut microbiomes. In this study, the gut microbiota datasets for these experimental animals were generated from fecal samples, which may serve as references to support their roles in comparative medicine.

## Results

### General comparison of human and five animal gut microbiota

The 16S rRNA sequence data were collected from the fecal samples from ten animals of each host species and from 151 Chinese people. The age and sex information were listed in supplementary Table S1. These sequence data were annotated to OTUs in a comparison with the Silva Database and the quantities of reads for each sample were provided in supplementary table about reads per sample. About 99.96% of the OTUs were annotated in the Silva Database (7151/7154). The annotation rates were 95.72% at the phylum level, 92.49% at the class level, 86.75% at the order level, 79.30% at the family level, 50.15% at the genus level, and 14.76% at the species level. The predominant phyla were Firmicutes, Actinobacteria, Bacteroidetes, Proteobacteria, and Fusobacteria (Table 1); the predominant classes were Clostridia, Bacilli, and Bacteroidia; the predominant orders were Clostridiales, Lactobacillales, and Bacteroidales; the predominant families were Lachnospiraceae, Bifidobacteriaceae, and Bacteroidaceae; and the predominant genera were *Bifidobacterium*, *Bacteroides*, and *Blauti*a.

**Table 1:** Top 10 phyla in the microbiota of human and animals.

Taxonomy	Percents in different animal species (%)					
	Human	Ferret	Marmoset	Mini pig	Woodchuck	Tree shrew
Firmicutes	62.1929	63.3721	11.5014	49.6277	44.4335	40.6838
Actinobacteria	8.2447	1.3214	49.5923	0.446	3.0154	3.4616
Bacteroidetes	21.3712	1.9017	28.4872	39.1893	35.9774	38.8865
Proteobacteria	5.6115	17.3624	6.2044	1.6604	5.1319	5.6852
Fusobacteria	0.1362	11.2208	2.0445	0.004	7.0467	9.9859
Verrucomicrobia	0.8095	0.0477	0.2754	0.1041	0.0188	0.0022
unidentified_Bacteria	0.2609	0.0739	1.4799	0.1271	3.9638	0.2519
Tenericutes	1.0903	4.1376	0.0042	2.0878	0.0551	0.286
Spirochaetes	0.0047	0.0381	0.0084	5.0001	0.0606	0.5325
Nitrospirae	0.0313	0.0099	0	0.001	0.0002	0.0005
Others	0.2469	0.5145	0.4022	1.7526	0.2967	0.2237

**Figure 1:**
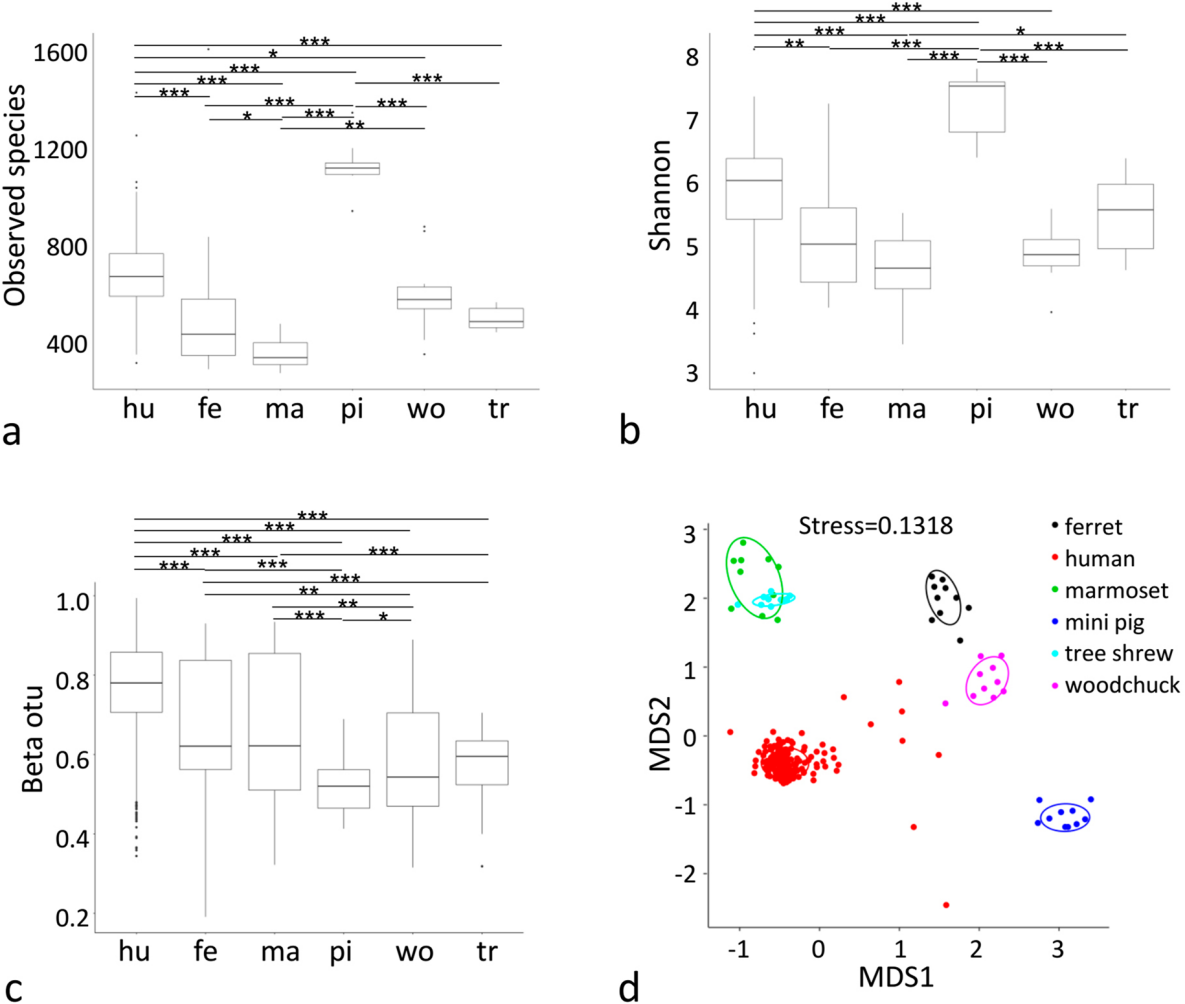
Statistical differences in the gut microbiota from human and five animal species. α-diversity estimates of the microbiota of human and five animal species based on OTU counts are shown as the numbers of observed bacterial species **(a)** and Shannon index **(b)**. β-Diversity is shown as beta OTU **(c)**. The Wilcox tests between groups are given. Significant differences areindicated with one asterisk (*, Wilcox p-value < 0.05), two asterisks (**, Wilcox p-value < 0.01) or three asterisks (***, Wilcox p-value < 0.001). Multi-dimensional scaling analysis result is shown in Fig. 2d.

The α-diversity of the gut microbiota of humans and five animal species are shown in Fig. 1a. There were significant differences in the bacterial species observed between humans and ferrets, marmosets, mini pigs, or tree shrews (Observed species, wilcox, p < 0.001), and between humans and woodchucks (Observed species, wilcox, p = 0.0425). More bacterial species were observed in the gut microbiota of the mini pig than in the human microbiota. Fewer bacterial species were observed in the gut microbiota of the ferret, marmoset, woodchuck, and tree threw than in the human microbiota (Fig. 1a). The Shannon index differed significantly between the human microbiota and those of the marmoset, mini pig, and woodchuck (Shannon, wilcox, p < 0.001), and between human and ferret (p = 0.0032) (Fig. 1b).

The β-diversity of the gut microbiota from the humans and five animal species is shown in Fig. 1c. There were significant differences in the gut microbiota of humans and those of the five animal species (β-diversity index on bray_curtis, wilcox, p < 0.001). β-Diversity was higher in the human gut microbiota than in those of the five animal species. The significant differences between the human microbiota and those of the other hosts can be visualized in multi-dimensional scaling analysis (MDS, Fig. 1d).

### Core and host specific microbiota of the five animal species

#### Human core microbiota

**Figure 2:**
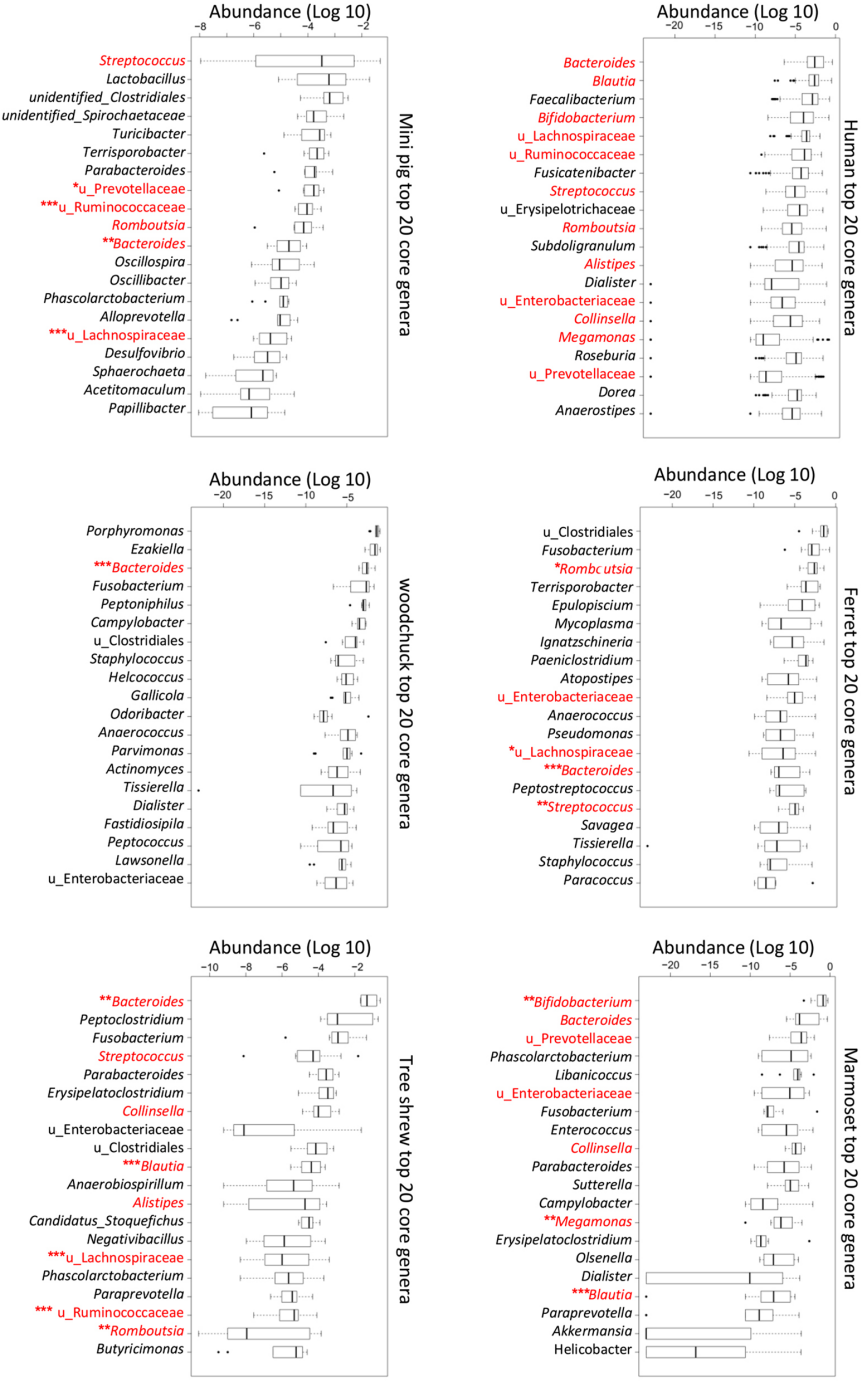
The core genera in the gut microbiota of human and five animal species. Comparison of the core genera in the gut microbiota. The top 20 genera of frequency in the gut microbiota of human and the five animal species are listed with their abundance (Log 10). Those shared genera between human and animal species are marked in red. The shared genera with significantly different abundances between human and animal species are indicated with one asterisk (*, Wilcox p-value < 0.05), two asterisks (**, Wilcox p-value < 0.01) or three asterisks (***, Wilcox p-value < 0.001).

The top 10 phyla in the human microbiota and those of the other five species are shown in Table 1, and the top 40 genera are shown in supplementary Table S2. In humans, the predominant phyla were Firmicutes, Bacteroidetes, Actinobacteria, and Proteobacteria. The core genera in the human gut microbiota were *Bacteroides, Blautia*, *Faecalibacterium*, *Bifidobacterium*, unidentified Lachnospiraceae, unidentified Ruminococcaceae, *Fusicatenibacter*, *Streptococcus*, unidentified Erysipelotrichaceae, *Romboutsia*, *Subdoligranulum*, *Alistipes*, *Dialister*, unidentified Enterobacteriaceae*, Collinsella*, *Megamonas*, *Roseburia,* unidentified Prevotellaceae, *Dorea*, *Anaerostipes*, *Lactococcus,* unidentified Clostridiales, and *Holdemanella*. The top 20 core genera in the gut microbiota from human and the five animal species are shown in Fig. 2, in which the human gut microbiota was used as the reference in the comparative analysis.

### Ferret microbiota

The proportion of Firmicutes was similar in the gut microbiota of the ferret and humans (63.37% vs 62.19%, respectively) at the phylum level. There were fewer Actinobacteria (1.32% vs 8.24%, respectively, *t* test, P < 0.001), Bacteroidetes (1.90% vs 21.37%, respectively, *t* test, P < 0.001), and Verrucomicrobia (0.05% vs 0.81%, respectively, *t* test, P < 0.05) in the gut microbiota of the ferret than in that of the human, but more Proteobacteria (17.36% vs 5.61%, respectively, *t* test, P < 0.05) and Fusobacteria (11.22% vs 0.14%, respectively, *t* test, P < 0.05).

A comparison of the linear discriminant analysis (LDA) effect size (LEfSe) of the human and ferret microbiota was performed [LDA score (log10) > 4.0]. Compared with the human gut microbiota, there were fewer *Bacteroides*, *Blautia*, *Faecalibacterium*, *Bifidobacterium*, *Fusicatenibacter*, and some unidentified members of the families Ruminococcaceae and Lachnospiraceae at the genus level, but more unidentified Clostridiales, *Fusobacterium*, *Romboutsia*, *Terrisporobacter*, *Epulopiscium, Mycoplasma*, *Ignatzschineria*, and *Paeniclostridium.* At the family level, there were more members of the Carnobacteriaceae in the ferret gut microbiota. Those ferret specific genera can be detected with Metastat (Supplementary Fig. S1).

#### Marmoset microbiota

There were fewer Firmicutes (11.50% vs 62.19%, respectively, *t* test, P < 0.001) and more Actinobacteria (49.59% vs 8.24%, respectively, *t* test, P < 0.05) and Tenericutes (1.09% vs 0.004%, respectively, *t* test, P < 0.001) in the gut microbiota of the marmoset than in human microbiota. In the marmoset gut microbiota, at the genus level, there were fewer unidentified Erysipelotrichaceae, *Fusicatenibacter*, *Blautia*, *Faecalibacterium*, and some unidentified Ruminococcaceae and Lachnospiraceae, but more *Bifidobacterium*, *Libanicoccus*, *Fusobacterium*, *Enterococcus*, *Phascolarctobacterium*, and unidentified Prevotellaceae and Enterobacteriaceae than in the human microbiota. Those marmoset specific genera can be detected with Metastat (Supplementary Fig. S2).

#### Mini pig microbiota

There were fewer Firmicutes (49.62% vs 62.19%, respectively, *t* test, P < 0.05), Actinobacteria (0.45% vs 8.24%, respectively, *t* test, P < 0.001), and Proteobacteria (1.66% vs 5.61%, respectively, *t* test, P < 0.001), but more Bacteroidetes (39.19% vs 21.37%, respectively, *t* test, P < 0.001) and Spirochaetes (5.00% vs 0.005%, respectively, *t* test, P < 0.001) in the gut microbiota of the mini pig than in the human microbiota. In the mini pig gut microbiota, at the genus level, there were fewer *Bifidobacterium*, *Bacteroides*, *Fusicatenibacter*, *Blautia*, *Faecalibacterium*, and some unidentified Erysipelotrichaceae and Lachnospiraceae, but more *Lactobacillus*, *Terrisporobacter*, *Turicibacter*, and some unidentified Clostridiales and Spirochaetaceae. Those mini pig specific genera can be detected with Metastat (Supplementary Fig. S3).

#### Woodchuck microbiota

There were fewer Firmicutes (44.43% vs 62.19%, respectively, *t* test, P < 0.05) and Actinobacteria (3.02% vs 8.24%, respectively, *t* test, P < 0.001), and more Bacteroidetes (35.98% vs 21.37%, respectively, *t* test, P < 0.05), and Fusobacteria (7.05% vs 0.14%, respectively, *t* test, P < 0.05) in the gut microbiota of the woodchuck than in the human microbiota. In the woodchuck gut microbiota, at the genus level, there were fewer *Bifidobacterium*, *Fusicatenibacter*, *Blautia*, *Faecalibacterium*, and some unidentified Erysipelotrichaceae, Lachnospiraceae, and Ruminococcaceae, but more *Porphyromonas*, *Ezakiella*, *Fusobacterium*, *Peptoniphilus*, and *Campylobacter*. Those woodchuck specific genera can be detected with Metastat (Supplementary Fig. S4).

### Tree shrew microbiota

There were fewer Firmicutes (40.68% vs 62.19%, respectively, *t* test, P < 0.05), and more Bacteroidetes (38.89% vs 21.37%, respectively, *t* test, P < 0.05) and Fusobacteria (9.99% vs 0.14%, respectively, *t* test, P < 0.05) in the gut microbiota of the tree shrew than in the human microbiota.

In the tree shrew gut microbiota, at the genus level, there were fewer *Bifidobacterium*, *Fusicatenibacter*, *Blautia*, *Faecalibacterium*, and some unidentified Lachnospiraceae and Ruminococcaceae, but more *Bacteroides*, *Peptoclostridium*, *Fusobacterium*, *Erysipelatoclostridium*, and *Parabacteroides*. Those tree shrew specific genera can be detected with Metastat (Supplementary Fig. S5).

### Clustering of the gut microbiota mirrors the host taxonomic phylogeny

**Figure 3:**
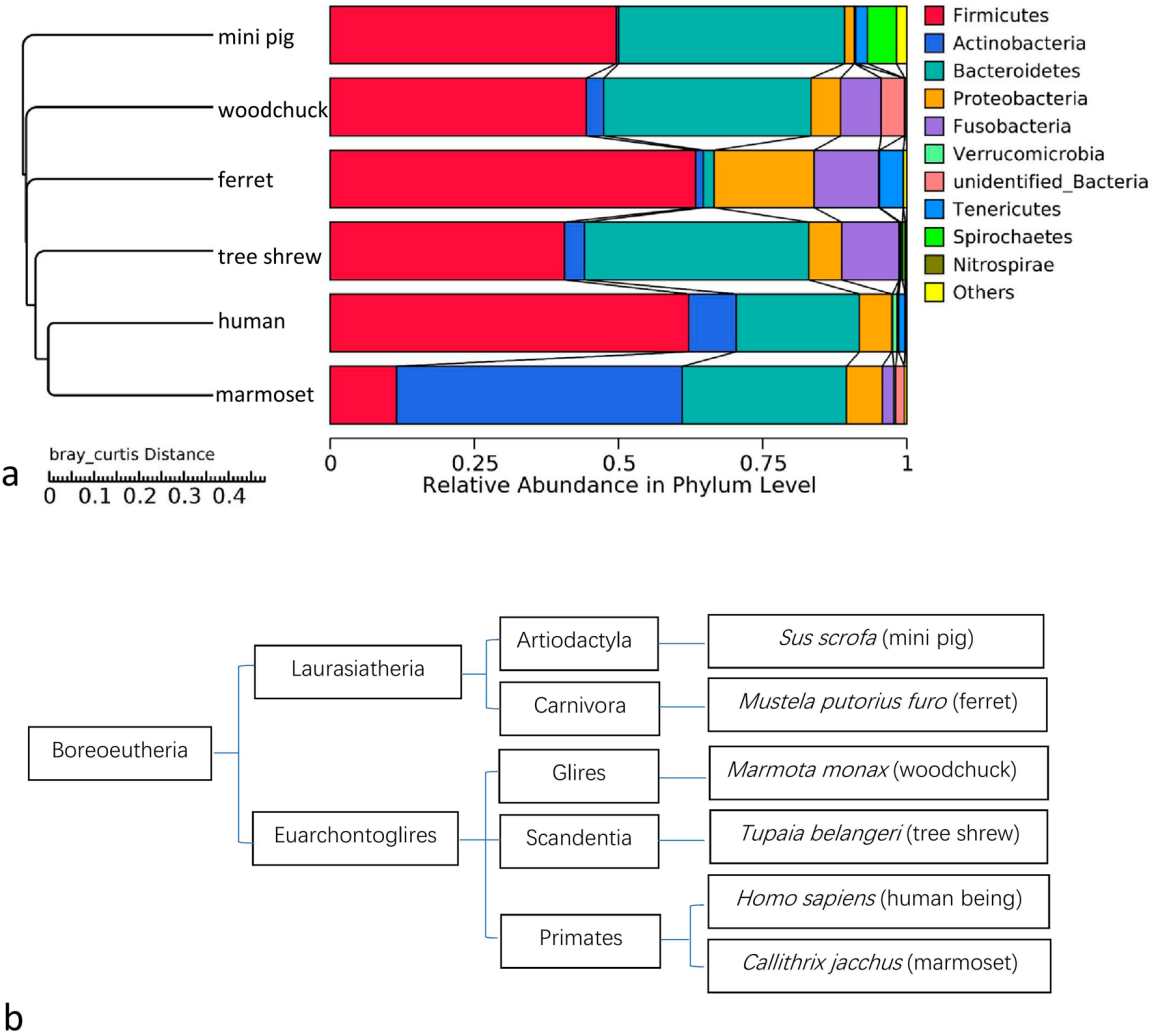
Gut microbiota clustering tree mirrors the taxonomic tree of the hosts. **(a)** Microbiota profile clustering based on *bray_curtis* dissimilarities is set to form an OTU hierarchical clustering tree of the overall grouped distribution of microbial species in the gut of each host species. The legend boxes list the 10 most abundant taxa at the phylum level. **(b)**Taxonomic tree of the six host species in this study is generated based on the taxonomy databases in National Center for Biotechnology Information (NCBI).

The OTUs from all the samples were used to develop a hierarchical clustering tree with UPGMA, and the average linkages were used to interpret the *bray_curtis* distance matrix between the samples (Supplementary Fig. S6). Most of the samples from the same species clustered together. The patterns of community similarity on the hierarchical OTU clustering tree (Fig. 3a) was similar to the mammalian phylogenetic tree (Fig. 3b). The overall distribution of the gut microbial species in the gut mirrors their host taxonomic phylogeny.

**Figure 4:**
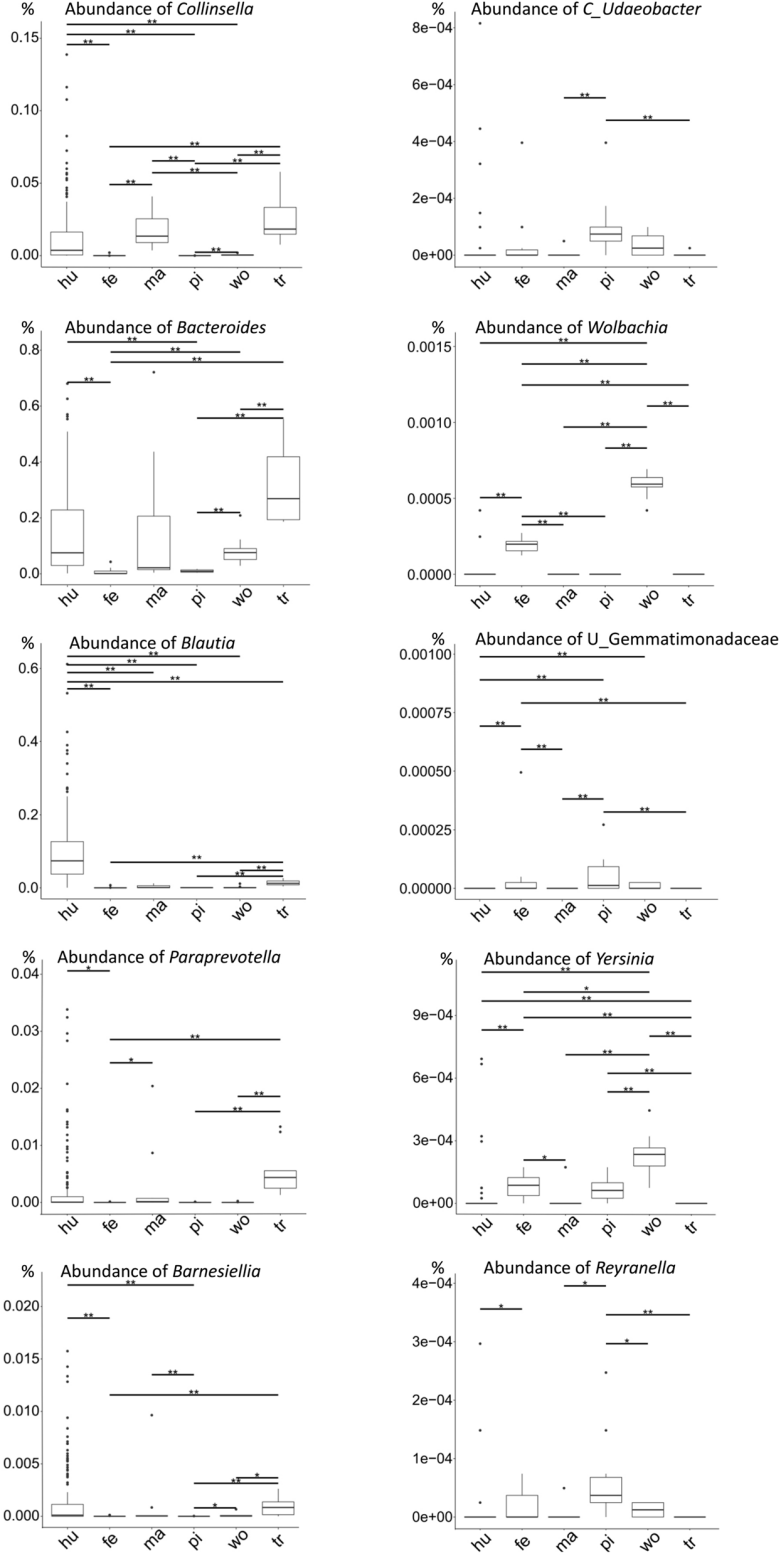
Host phylogenetic related genera detected with Metastat. The host phylogenetic related genera are detected with Metastat. The genera of *Collinsella*, *Bacteroides*, *Blautia*, *Paraprevotella*, and *Barnesiella* predominately existed in the microbiota of human (hu), marmoset (ma), and tree shrew (tr). The *Candidatus Udaeobacter*, *Wolbachia*, unidentified Gemmatimonadaceae, *Yersinia*, and *Reyranella* existed in the microbiota of ferret (fe), mini pig (pi), and woodchuck (wo). The abundance of each genus is shown as value in percent (%). The statistic differences of the genera abundances from different host species are indicated with one asterisk (*, Wilcox p-value < 0.05) or two asterisks (**, Wilcox p-value < 0.01).

To test the hypothesis that the relationships between the gut microbiota of the human, marmoset, and tree shrew are closer than the others, the comparative Metastat results were screened and the genera *Collinsella*, *Bacteroides*, *Blautia*, *Paraprevotella*, and *Barnesiella* were mostly identified in the microbiota of the human, marmoset, and tree shrew (Fig. 4).

### Influence of diet on the gut microbiota of the five experimental animal species

**Figure 5:**
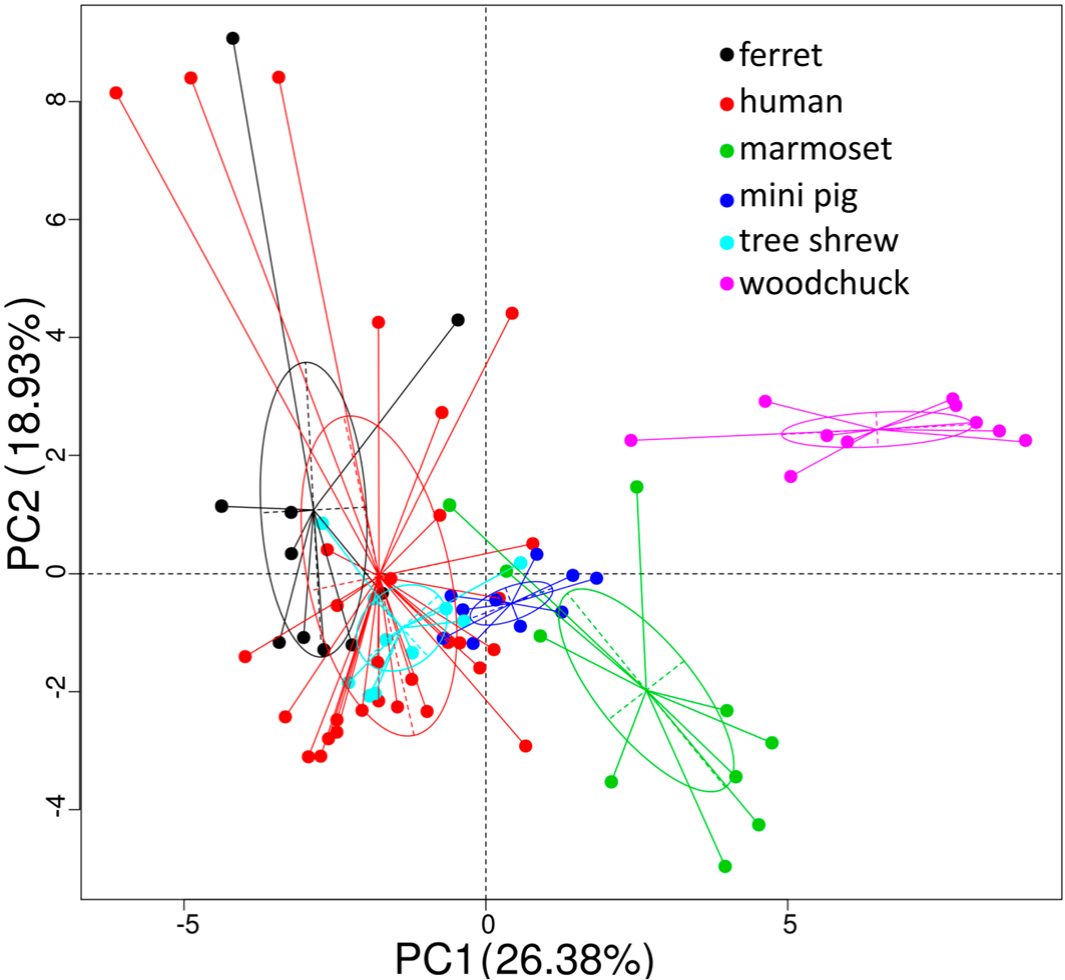
Principal components analysis of PICRUSt functional predictions. Functional prediction is made with PICRUSt. A principal components analysis (PCA) is used to compare of the functions of the gut microbiota, and the predicted data in level 2 of KEGG^25^ are shown.

The results described above imply that the codiverdification of the gut microbiota with their hosts. We used PICRUSt to functionally compare the gut microbiota. A PCA based on the KEGG^25^ database was used to compare the functional compositions of the microbiota of humans and the five animal species tested (Fig. 5). The gut microbiota of woodchuck were separated from that of human, ferret, tree shrew, and mini pig.

## Discussion

In comparative medical research, a better understanding of the similarities and differences between human and animal models improves the translational utility of the information obtained from animal experiments. The evolutionary histories, genetic backgrounds, and anatomical structures of mice and rats have been thoroughly evaluated and systematically compared with those of humans^9^. The gut microbiomes of mice and rats have also been measured to explore the relationships between the microbiota and host health^26^. A consensus has been reached about the importance of the gut microbiota to the health of the host^27^.

In this study, the gut microbiota of five recently used model animals were analyzed with 16S rRNA sequencing to detect their core bacterial genera. Differential genera were found in the core genera of each host species which were showed in Fig. 2, Fig. 4 and in the supplementary figures. The genus *Collinsella* occurred in the gut microbiota of human, marmoset and tree shrew, whereas it was limited in the gut microbiota of the ferret, mini pig, and woodchuck. Although the genus *Bacteroides* was present in the gut microbiota of all six host species, it was much more abundant in those of the human, marmoset, and tree shrew. The genus *Blautia* occurred in the core human gut microbiota, and also in those of the marmoset and tree shrew. The genera *Paraprevotella* and *Barnesiella* appeared in the core gut microbiota of both the human and tree shrew. While the trace genera *Candidatus Udaeobacter*, *Wolbachia*, *Yersinia*, *Reyranella*, and unidentified Gemmatimonadaceae were detected in the microbiota of ferret, mini pig, and woodchuck (Fig. 4). In an evolutionary view, marmoset belongs to the order of primates, and is the closest relative of human (Fig. 3b). Tree shrew, a member of the superorder Euarchontoglires, is a close relative of the primates. The genera of *Collinsella*, *Bacteroides*, *Blautia*, *Paraprevotella*, and *Barnesiella* are more likely maintained in these phylogenetic related host species.

Change in ecological niche is believed to influence the composition of the host microbiota^28,29^. The marmoset is a New World primate that originated on the South American continent^30^. Therefore, the marmoset occurs at a great geographic distance from the Chinese human volunteers used as the reference in this study. The tree shrew is another distant relative of *Homo sapiens*, located geographically in south Asia^31^. The marmoset and tree shrew are neither livestock nor pets, and they do not share a close ecological niche with humans. However, in the present study, closer relationships between the gut microbiota of human and marmosets or tree shrew were detected when the *bray_curtis* distances were compared with the other three animal species (Fig. 3). The phylogenetic lineage of the hosts can have significant influence on their microbiota, and more closely related species exhibit more similar gut microbiota^32^. The gut microbiota have been believed to co-diversify along with their hosts^33,34^. The gut microbiota variation has been corelated with the host genetics in the same species^35^. The microbiota can be associated with host genetics. In a recent study, the gut microbiota of 285 pigs in the same farm under intensive conditions had been corelated with the pig genome^36^.

Diet is believed to affect the diversity of the gut microbiota^37^, and omnivorous hosts should have a more diverse microbiota than carnivores^38–40^. In this study, the mini pigs were raised with formula fodder similar to those of the other animals. But more bacterial species were detected in the mini pig gut microbiota than in other hosts. And the core genera of the other pigs were also detected in the experimental mini pigs^38^. The experimental mini pig colony was bred from commercially farmed pigs, and their omnivorous breeding history has also left a trace in the gut microbiota of the mini pigs.

In the functional predication analysis of the gut microbiota, The PCA distances at KEGG level 2 separate woodchuck from the other five hosts (Fig. 5), which may reflect differences in their dietary habits. The woodchuck was the only herbivorous animal included in the study. Therefore, these data support, at least to some extent, the notion that the dietary composition greatly affects the microbiome in the host gut.

Clues of opportunistic pathogens were also detected in the gut microbiota analysis. The health status of the animals in the study was recorded as good. However, the genus *Mycoplasma* was detected in three female ferrets out of the ten sampled ones. Although the mycoplasma culture results were negative for the corresponding frozen samples (data not shown). Some pathogenic organisms like the genera of *Yersinia* were also detected. More attention should be given for these pathogens in their health surveillance. This dataset will be used to determine the targeted pathogens in their health surveillances programs.

The biological properties of experimental animals are extremely important in comparative preclinical medicine, and the gut microbiota has been regarded as an emerging organ that should not be ignored^27^. Both evolutionary phylogeny and diet may affect the gut microbiota. The comparative microbiota data from these experimental animals may contribute to their future usage in comparative medical research.

## Research limitation

The gut microbiota is much dynamic, which may be influenced by the diet, environment, and host status. In this study, the sample size is small, and the finding in this study could be specific to the samples. The gut microbiota may vary when samples are taken from different animals or the same animal at different seasons or animals of the different populations in the same species. Further surveillance may contribute to understand the influence of evolutionary phylogeny and diet on the gut microbiota of these animals.

## Methods

### Information about the animals included in this study

All the animals included in this study were raised at the Institute of Laboratory Animal Science, CAMS&PUMC. The ferret, marmoset, woodchuck, and tree shrew were raised in separated barrier facilities, under controlled temperature at 20–26 ℃ and humidity at 40–70%. and the mini pigs were raised in a semibarrier facility: with controlled temperature at 20–26 ℃ in their bed rooms, and with free access to the atmosphere in their living rooms. The animals in different facilities were raised by different breeders, and there were enough distances between the facilities to avoid the possible pathogens transmission. The health status of these animals was monitored by the veterinarians and all the animals colonies were in good health status during samples collection.

All the animals were fed with commercial formula fodders. The ingredients of the ferret formula fodder were fresh chicken, peruvian fishmeal, chickenmeal, corn, flour, soybean meal, and compound microorganisms. A formula fodder for dogs was used for the marmosets and tree shrews, and contained beet pulp, fishmeal, chickenmeal, corn, and animal fat. The ingredients of the pig formula fodder were corn, flour, soybean meal, and bran. A formula fodder for rabbits was used for the woodchucks, and contained alfalfa powder, corn, flour, soybean meal, and yeast powder. Composite minerals and vitamins were included in all the formula fodders. The component ratios of these fodders are listed in Supplementary Table S3.

### Fecal samples collection

During the sampling time in June, 2018, the feces of the animals in the morning were observed and the middle parts of the fresh animal feces were collected at a volume of 0.5 g with sterile sticks and 15 mL centrifuge tubes to avoid the contamination with bedding and urine. Ten animals from each animal species were sampled and one fecal sample was taken from each animal for subsequent analysis. The age and sex information about the sampled animals are summarized in Supplementary Table S1. The samples were frozen in sterile containers and stored at − 80 °C before DNA extraction.

Reference human fecal samples were obtained from 151 healthy adult volunteers in Beijing area during their physical health examination. And informed consent had been obtained from all the participates in the study. The sampling procedure were based on the clinical guideline in the hospital. The samples were also frozen in sterile containers and stored at − 80 °C before DNA extraction.

### DNA extraction

Total genomic DNA was extracted from the fecal samples mentioned above with the CTAB/SDS method^41^. The extracted DNA was qualified by 1% agarose gel electrophoresis and the OD 260/280 ratio measurement. All of the DNA samples were stored at − 20 °C until further processing.

### 16S rRNA sequencing

The V4 region of 16S rRNA was amplified with the V4-specific primers 515F (5′-GTGCCAGCMGCCGCGGTAA-3′) and 806R (5′-GGACTACHVGGGTWTCTAAT-3′)^42^. The PCR was conducted for 35 cycles under the following conditions: denaturation at 95 °C for 60 s, annealing at 57 °C for 45 s and extension at 72 °C for 60 s. The PCR products were detected on 2% agarose gel electrophoresis and purified with the GeneJET Gel Extraction Kit (Thermo Scientific, Waltham, MA). The purified PCR products were quantified to the same amount for each sample to generate the libraries. The sequence libraries were generated with the Ion Plus Fragment Library Kit (Thermo Scientific). The libraries were quantified with the Qubit 2.0 Fluorometer (Thermo Scientific), and sequenced on the Ion S5 XL platform, generating single-end reads.

## Data analysis

### Operational taxonomic units (OTUs) annotation

The raw reads were quality filtered under specific filtering conditions according to the Cutadapt (v1.9.1)^43^ quality control process to obtain high-quality clean reads. The reads were compared with the Silva Database^44^ using the UCHIME algorithm^45^ to detect chimeric sequences, which were removed to generate clean reads. Sequences with ≥ 97% similarity were assigned to the same OTU. A representative sequence for each OTU was screened for further annotation^46^. The quantities of reads for each sample were provided in supplementary materials. The Silva Database was used to annotate the sequences with taxonomic information using the Mothur algorithm^47^. The OTU abundance information was normalized using a standard of sequence number corresponding to the sample with the least sequences. A multiple-sequence alignment was constructed with the MUSCLE software (version 3.8.31)^48^ for OTU clustering.

### α- and β-diversity analyses

α-diversity is a measure of the complexity of the species in one sample, and includes the number of species observed and the Shannon index. These indices were calculated for our samples with QIIME (version 1.7.0)^49^ and displayed with the R software (version 2.15.3). A β-diversity analysis was used to evaluate the differences in the species complexities of the samples. β-Diversity was calculated as the *bray_curtis* dissimilarity distances. To assess the beta diversity of all samples, the OTU table was normalized using the cumulative sum scaling (CSS) normalization procedure, the *bray_curtis* distance based on the normalized OTU table was set and a multi-dimensional scaling analysis was performed^50^.

### Microbiotal differences

The differences in the microbiota of the animal species were tested with Metastat^51^ in the R software and with the LEfSe software (LEfSe 1.0)^52^. Unweighted pair-group method with arithmetic mean (UPGMA) clustering was used as the hierarchical clustering method to interpret the distance matrix using average linkages, with the QIIME software. PICRUSt was used to predict the functional gene contents in the gut microbiota based on the taxonomy established with the Greengenes reference database ^53,54^. A principal components analysis (PCA) was performed at levels 2 of the Kyoto Encyclopedia of Genes and Genomes (KEGG)^25^.

### Ethics approval

In this study, we used microbiotal data from humans as the reference data for comparison with those of animals. The use of human samples was approved by the Ethics Committee of Peking Union Medical College Hospital (S-K478, Beijing, China) and the research had obtained consent from all the participates in the study.

The animal health surveillance protocols used in this study were approved by the Institutional Animal Care and Use Committee of the Institute of Laboratory Animal Science (XZG2017-001, Beijing, China).

The methods in the current research were carried out in accordance with the guideline for human medical research in Peking Union Medical College and the guideline for animal research in Institute of Laboratory Animal Science.

## Supplementary information

Supplementary Information 1.

Supplementary Information 2.

## Data Availability

The sequence data are available in the SRA database under accession number PRJNA524212 (https://www.ncbi.nlm.nih.gov/sra/PRJNA524212).

## References

[1] O'Hara AM, Shanahan F (2006). The gut flora as a forgotten organ. EMBO Rep..

[2] Le Chatelier E (2013). Richness of human gut microbiome correlates with metabolic markers. Nature.

[3] Cani PD (2008). Changes in gut microbiota control metabolic endotoxemia-induced inflammation in high-fat diet-induced obesity and diabetes in mice. Diabetes.

[4] Zhang X (2015). The oral and gut microbiomes are perturbed in rheumatoid arthritis and partly normalized after treatment. Nat. Med..

[5] Jie Z (2017). The gut microbiome in atherosclerotic cardiovascular disease. Nat. Commun..

[6] Lagkouvardos I (2016). The mouse intestinal bacterial collection (miBC) provides host-specific insight into cultured diversity and functional potential of the gut microbiota. Nat. Microbiol..

[7] Clayton JB (2016). Captivity humanizes the primate microbiome. Proc. Natl. Acad. Sci. U S A.

[8] Li, X. *et al.* Establishment of a *Macaca fascicularis* gut microbiome gene catalog and comparison with the human, pig, and mouse gut microbiomes. *Gigascience* **7**, 10.1093/gigascience/giy100 (2018). 10.1093/gigascience/giy100 PMC6137240 30137359

[9] Nguyen TL, Vieira-Silva S, Liston A, Raes J (2015). How informative is the mouse for human gut microbiota research?. Dis. Model Mech..

[10] Xu L (2014). Novel avian-origin human influenza A(H7N9) can be transmitted between ferrets via respiratory droplets. J. Infect. Dis..

[11] Schwerin SC (2017). Establishing the ferret as a gyrencephalic animal model of traumatic brain injury: Optimization of controlled cortical impact procedures. J. Neurosci. Methods.

[12] Iwatsuki-Horimoto K (2018). The marmoset as an animal model of influenza: Infection with A(H1N1)pdm09 and highly pathogenic A(H5N1) viruses via the conventional or tracheal spray route. Front. Microbiol..

[13] Kishi N, Sato K, Sasaki E, Okano H (2014). Common marmoset as a new model animal for neuroscience research and genome editing technology. Dev. Growth Differ..

[14] Okano H, Hikishima K, Iriki A, Sasaki E (2012). The common marmoset as a novel animal model system for biomedical and neuroscience research applications. Semin. Fetal Neonatal Med..

[15] Prins NW (2017). Common marmoset (*Callithrix jacchus*) as a primate model for behavioral neuroscience studies. J. Neurosci. Methods.

[16] Pajarillo EA (2014). Pyrosequencing-based analysis of fecal microbial communities in three purebred pig lines. J. Microbiol..

[17] Pedersen R (2013). Characterisation of gut microbiota in Ossabaw and Gottingen minipigs as models of obesity and metabolic syndrome. PLoS ONE.

[18] Ramos L (2017). The minipig as an animal model to study *Mycobacterium tuberculosis* infection and natural transmission. Tuberculosis (Edinb).

[19] Vamathevan JJ (2013). Minipig and beagle animal model genomes aid species selection in pharmaceutical discovery and development. Toxicol. Appl. Pharmacol..

[20] Menne S, Cote PJ (2007). The woodchuck as an animal model for pathogenesis and therapy of chronic hepatitis B virus infection. World J. Gastroenterol..

[21] Wang BJ (2011). Establishing a new animal model for hepadnaviral infection: susceptibility of Chinese Marmota-species to woodchuck hepatitis virus infection. J. Gen. Virol..

[22] Amako Y (2010). Pathogenesis of hepatitis C virus infection in *Tupaia belangeri*. J. Virol..

[23] Li R (2018). Tree shrew as a new animal model to study the pathogenesis of avian influenza (H9N2) virus infection. Emerg. Microbes Infect..

[24] Ye L (2016). Tree shrew as a new animal model for the study of lung cancer. Oncol. Lett..

[25] Kanehisa M, Goto S (2000). KEGG: Kyoto encyclopedia of genes and genomes. Nucleic Acids Res..

[26] Backhed F, Manchester JK, Semenkovich CF, Gordon JI (2007). Mechanisms underlying the resistance to diet-induced obesity in germ-free mice. Proc. Natl. Acad. Sci. U S A.

[27] Haluzik M, Kratochvilova H, Haluzikova D, Mraz M (2018). Gut as an emerging organ for the treatment of diabetes: Focus on mechanism of action of bariatric and endoscopic interventions. J. Endocrinol..

[28] Delsuc F (2014). Convergence of gut microbiomes in myrmecophagous mammals. Mol. Ecol..

[29] Sanders JG (2015). Baleen whales host a unique gut microbiome with similarities to both carnivores and herbivores. Nat. Commun..

[30] Antunes SG (1998). The common marmoset: A new world primate species with limited Mhc class II variability. Proc. Natl. Acad. Sci. U S A.

[31] Zhou X, Sun F, Xu S, Yang G, Li M (2015). The position of tree shrews in the mammalian tree: Comparing multi-gene analyses with phylogenomic results leaves monophyly of Euarchonta doubtful. Integr. Zool..

[32] Nishida AH, Ochman H (2018). Rates of gut microbiome divergence in mammals. Mol. Ecol..

[33] Moran NA (2006). Symbiosis. Curr. Biol..

[34] Nishida AH, Ochman H (2019). A great-ape view of the gut microbiome. Nat. Rev. Genet..

[35] Turpin W (2016). Association of host genome with intestinal microbial composition in a large healthy cohort. Nat. Genet..

[36] Crespo-Piazuelo D (2019). Association between the pig genome and its gut microbiota composition. Sci. Rep..

[37] Hale VL (2018). Diet versus phylogeny: A comparison of gut microbiota in captive colobine monkey species. Microb. Ecol..

[38] Holman, D. B., Brunelle, B. W., Trachsel, J. & Allen, H. K. Meta-analysis to define a core microbiota in the swine gut. *mSystems* **2**, 10.1128/mSystems.00004-17 (2017). 10.1128/mSystems.00004-17 PMC5443231 28567446

[39] Ley RE (2008). Evolution of mammals and their gut microbes. Science.

[40] Muegge BD (2011). Diet drives convergence in gut microbiome functions across mammalian phylogeny and within humans. Science.

[41] Archer SD, McDonald IR, Herbold CW, Lee CK, Cary CS (2015). Benthic microbial communities of coastal terrestrial and ice shelf Antarctic meltwater ponds. Front. Microbiol..

[42] Hugerth LW (2014). DegePrime, a program for degenerate primer design for broad-taxonomic-range PCR in microbial ecology studies. Appl. Environ. Microbiol..

[43] Martin, M. CUTADAPT removes adapter sequences from high-throughput sequencing reads. *EMBnet.journal* **17**, 10.14806/ej.17.1.200 (2011).

[44] Quast C (2013). The SILVA ribosomal RNA gene database project: Improved data processing and web-based tools. Nucleic Acids Res..

[45] Edgar RC, Haas BJ, Clemente JC, Quince C, Knight R (2011). UCHIME improves sensitivity and speed of chimera detection. Bioinformatics.

[46] Edgar RC (2013). UPARSE: Highly accurate OTU sequences from microbial amplicon reads. Nat. Methods.

[47] Schloss PD (2009). Introducing mothur: Open-source, platform-independent, community-supported software for describing and comparing microbial communities. Appl. Environ. Microbiol..

[48] Edgar RC (2004). MUSCLE: Multiple sequence alignment with high accuracy and high throughput. Nucleic Acids Res..

[49] Caporaso JG (2010). QIIME allows analysis of high-throughput community sequencing data. Nat. Methods.

[50] Paulson JN, Stine OC, Bravo HC, Pop M (2013). Differential abundance analysis for microbial marker-gene surveys. Nat. Methods.

[51] White JR, Nagarajan N, Pop M (2009). Statistical methods for detecting differentially abundant features in clinical metagenomic samples. PLoS Comput. Biol..

[52] Segata N (2011). Metagenomic biomarker discovery and explanation. Genome Biol..

[53] Langille MG (2013). Predictive functional profiling of microbial communities using 16S rRNA marker gene sequences. Nat. Biotechnol..

[54] DeSantis TZ (2006). Greengenes, a chimera-checked 16S rRNA gene database and workbench compatible with ARB. Appl. Environ. Microbiol..

