# Supplementary material for: A glance at the gut microbiota of five experimental animal species through fecal samples
Source: Sci Rep. 2020 Oct 6;10:16628. doi: 10.1038/s41598-020-73985-2 (PMC7538948; doi:10.1038/s41598-020-73985-2)
Supplement: Supplementary file 1 — Supplementary Information 1. [file 41598_2020_73985_MOESM1_ESM.docx]

**A glance at the gut microbiota of five experimental animal species through fecal samples**

Zhiguang Xiang, Hua Zhu, Bochao Yang, Hang Fan, Jianguo Guo, Jiangning Liu, Qi Kong, Qingfeng Teng, Haiquan Shang, Lei Su, Chuan Qin

**Table S1. The age and sex information of animals**

| **species** | **Animal code and their age sex information** | | | | | | | | | | |
| --- | --- | --- | --- | --- | --- | --- | --- | --- | --- | --- | --- |
| Tree Shrew | **code** | 17 | 20 | 5 | 19 | 21 | 16 | 15 | 12 | 18 | 118 |
|  | age | 1 | 6 | 1 | 2 | 2 | 2 | 1 | 1 | 1 | 4 |
|  | sex | M | F | M | M | M | M | F | F | F | F |
| Marmoset | code | s2-3 | s4-8 | s3-6 | s3-1 | s3-12 | s2-2 | s3-10 | s2-1 | s3-7 | s3-4 |
|  | age | 5 | 4 | 4 | 4 | 4 | 2 | 3 | 5 | 4 | 3 |
|  | sex | F | F | M | F | F | F | F | F | F | M |
| woodchuck | code | 2755F | 2745F | 2762F | 5270F | 3177F | 2746F | 2921F | 1701F | 2848F | 2722F |
|  | age | 5 | 4 | 2 | 1 | 2 | 5 | 3 | 1 | 5 | 3 |
|  | sex | M | M | F | F | M | F | M | M | F | F |
| Ferret | code | 0009F | 024F | 028F | 040F | 046F | 051F | 054F | 062F | 116F | 118F |
|  | age | 5 | 4 | 5 | 1 | 1 | 4 | 4 | 4 | 3 | 3 |
|  | sex | M | M | M | M | M | M | F | F | F | M |
| mini pig | code | P2 | P3 | P4 | P6 | P8 | P9 | P10 | P11 | P13 | P15 |
|  | age | 1 | 1 | 1 | 1 | 1 | 1 | 1 | 1 | 2 | 2 |
|  | sex | F | F | F | M | F | F | F | F | M | F |

Note：The **codes** listed in the table are animal codes which had been used in data analysis. The age unit is year. The F in sex table means female, and M means male.

**Table S2.** **The microbiota of human and animals in the top 40 genera**

| **Taxonomy** | **Percents in different species (%)** | | | | | |
| --- | --- | --- | --- | --- | --- | --- |
|  | **hu** | **fe** | **Ma** | **pi** | **wo** | **tr** |
| *Bifidobacterium* | 5.4061 | 0.1122 | 39.6111 | 0.1914 | 0.0507 | 0.1399 |
| *Bacteroides* | 14.7036 | 0.8344 | 15.639 | 1.0131 | 8.4934 | 31.2559 |
| *Blautia* | 10.2022 | 0.0846 | 0.3558 | 0.0541 | 0.1822 | 1.3815 |
| *Peptoclostridium* | 0.4178 | 0.0017 | 0.046 | 0 | 0.0049 | 16.9193 |
| *Lactobacillus* | 0.8758 | 0.1431 | 0.0111 | 5.2958 | 0.0638 | 0.0096 |
| *Lactococcus* | 1.0712 | 0.0304 | 0.004 | 0.0099 | 0.0326 | 0.1609 |
| *Fusobacterium* | 0.1293 | 11.1956 | 2.0263 | 0.0032 | 6.8907 | 7.6632 |
| *Faecalibacterium* | 8.4438 | 0.1075 | 0.0002 | 0.0292 | 0.0712 | 0.0007 |
| *Megamonas* | 1.3357 | 0.0205 | 0.7869 | 0.0012 | 0.0136 | 0.1839 |
| *Akkermansia* | 0.7867 | 0.0146 | 0.2742 | 0.0714 | 0.0124 | 0.001 |
| unidentified_Clostridiales | 1.0699 | 23.7079 | 0.0423 | 4.784 | 1.9012 | 1.9377 |
| *Ezakiella* | 0.0027 | 0.0764 | 0 | 0 | 20.4967 | 0 |
| *Porphyromonas* | 0.0105 | 0.2381 | 0.0027 | 0.0017 | 25.0726 | 0.0012 |
| *Enterococcus* | 0.6156 | 0.0428 | 1.8853 | 0.002 | 0.0344 | 0.1263 |
| *Weissella* | 0.253 | 0.0161 | 0.0074 | 0.0084 | 0.0087 | 0.0015 |
| *Streptococcus* | 2.2246 | 0.8158 | 0.2109 | 6.3475 | 0.4373 | 3.1185 |
| *Dialister* | 1.4099 | 0.0131 | 0.4302 | 0.0121 | 0.5397 | 0.002 |
| *Romboutsia* | 1.7484 | 8.1817 | 0.0025 | 1.6732 | 0.0072 | 0.5254 |
| unidentified_Enterobacteriaceae | 1.3822 | 1.4972 | 2.2925 | 0.1206 | 0.4561 | 2.2816 |
| *Rhodococcus* | 0.6018 | 0.0791 | 0.0012 | 0.0064 | 0.0007 | 0.0017 |
| *Ignatzschineria* | 0 | 3.6283 | 0 | 0.0005 | 0.0007 | 0 |
| **(Continue Table S2)**  **Taxonomy** | Percents in different species (%) | | | | | |
|  | hu | fe | Ma | pi | Wo | tr |
| *Subdoligranulum* | 1.6725 | 0.0351 | 0.0005 | 0.044 | 0.0072 | 0.0312 |
| unidentified_Prevotellaceae | 1.319 | 0.1731 | 4.0666 | 2.1672 | 0.1869 | 0.043 |
| *Holdemanella* | 1.014 | 0.0425 | 0 | 0.0072 | 0.0141 | 0 |
| *Roseburia* | 1.3212 | 0.0576 | 0.0007 | 0.2084 | 0.0148 | 0.0035 |
| unidentified_Erysipelotrichaceae | 2.0624 | 0.0806 | 0.1014 | 0.0079 | 0.0148 | 0.0494 |
| *Fusicatenibacter* | 2.4471 | 0.0077 | 0.0002 | 0.0025 | 0.0025 | 0 |
| *Sulfuricurvum* | 0.1632 | 0.0007 | 0 | 0.0005 | 0.0002 | 0 |
| *Alistipes* | 1.4393 | 0.0368 | 0.0146 | 0.0319 | 0.1654 | 1.1684 |
| *Mycoplasma* | 0.0013 | 4.12 | 0.0007 | 0.0017 | 0.0012 | 0.0952 |
| *Anaerostipes* | 1.199 | 0.0116 | 0.0005 | 0.0148 | 0.0017 | 0.0178 |
| unidentified_Ruminococcaceae | 3.1255 | 0.046 | 0.0072 | 1.8811 | 0.1464 | 0.5276 |
| *Alloprevotella* | 0.1845 | 0.0717 | 0.1884 | 0.6856 | 0.067 | 0.0176 |
| *Aeromonas* | 0.1957 | 0.0299 | 0 | 0.0002 | 0.0005 | 0 |
| *Parasutterella* | 0.5492 | 0.0116 | 0.0002 | 0 | 0.0252 | 0.0134 |
| *Terrisporobacter* | 0.0553 | 6.0751 | 0 | 2.5612 | 0.0079 | 0.0002 |
| unidentified_Lachnospiraceae | 3.3638 | 1.0791 | 0.1434 | 0.5568 | 0.0724 | 0.6789 |
| *Epulopiscium* | 0.0001 | 4.3121 | 0 | 0 | 0.0007 | 0 |
| *Catenibacterium* | 0.2652 | 0.0027 | 0.0002 | 0.0002 | 0.0087 | 0 |
| *Collinsella* | 1.368 | 0.0215 | 1.7439 | 0.001 | 0.0566 | 2.4065 |
| Others | 25.5627 | 32.9435 | 30.1019 | 72.2018 | 34.435 | 29.2353 |

**Table S3. The components of the formula fodders for animals**

| **component** | **Ferret** | **minipig** | **Marmoset, tree shrew** | **Woodchuck** |
| --- | --- | --- | --- | --- |
| protein | ≥34% | ≥16% | ≥30% | ≥17% |
| Crude fat | ≥16% | ≥2% | ≥20% | ≥3% |
| Crude fiber | ≤5% | ≤8% | ≤3% | 10-15% |
| Crude ash | ≤10% | ≤8% | ≤9% | ≤9% |
| Moisture | ≤10% | ≤12% | ≤10% | ≤11% |
| Calcium | ≥0.8% | 0.6-1.0% | 1.0-1.5% | 1.0-1.5% |
| Phosphorus | ≥1.0% | 0.5-0.8% | 0.8-1.2% | 0.5-0.8% |

Note: the commercial dog fodder was given to marmoset and tree shrew; the commercial rabbit fodder was given to woodchuck.


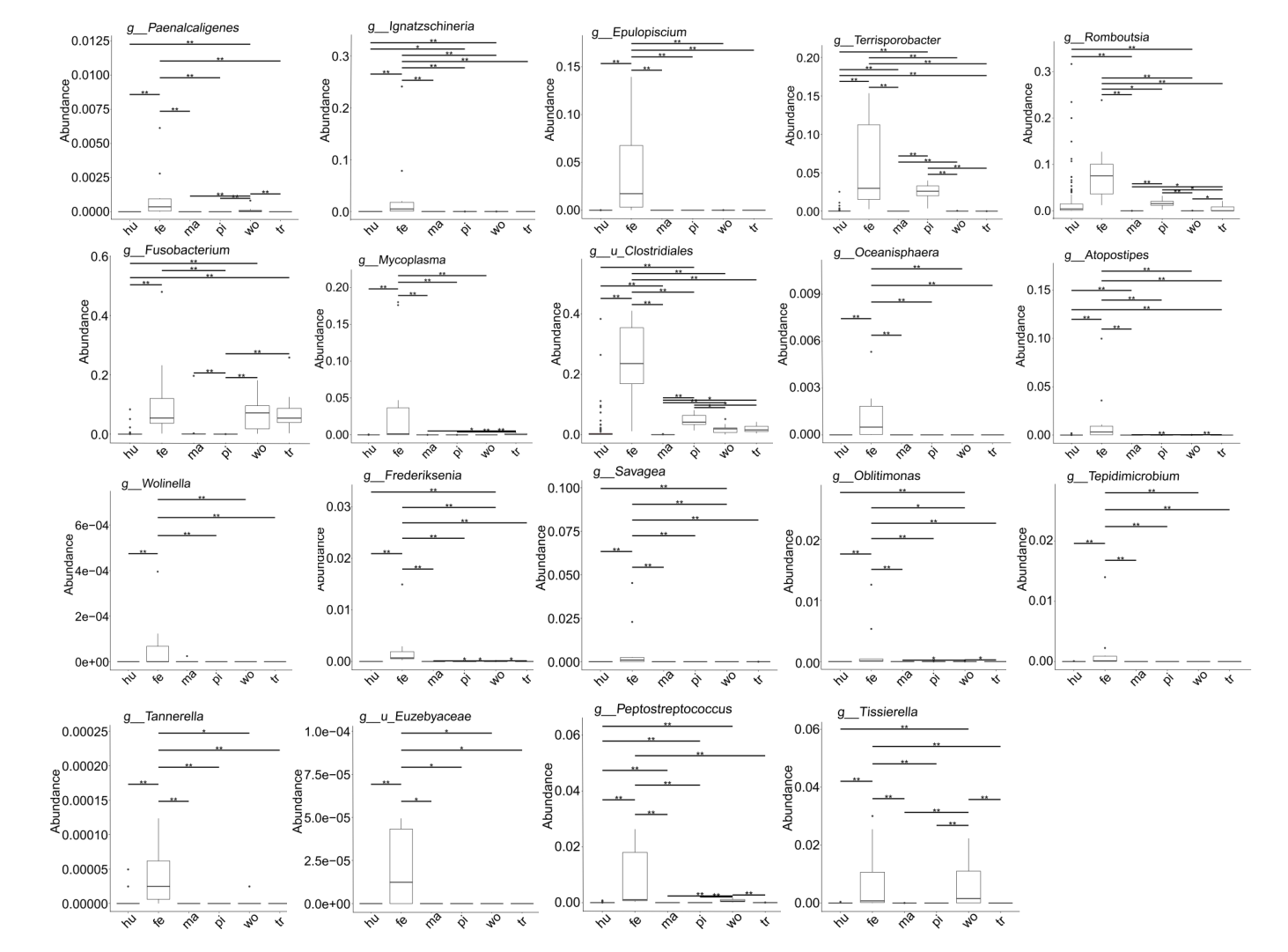


**Fig. S1 Host specific genera detected with Metastat in ferret** (abundance: frequency in gut microbiota %; ***,** Wilcox p <0.05; ****,** p<0.01)


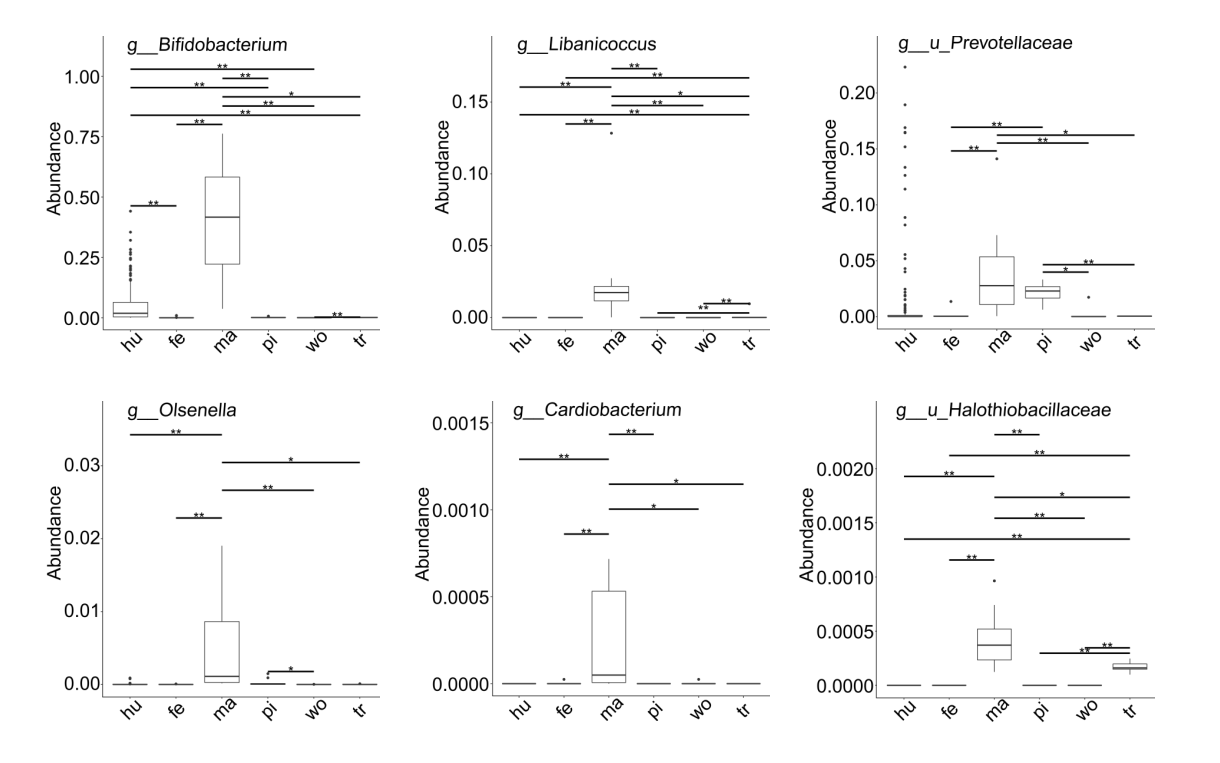


**Fig. S2 Host specific genera detected with Metastat in marmoset** (abundance: frequency in gut microbiota %; ***,** Wilcox p <0.05; ****,** p<0.01)


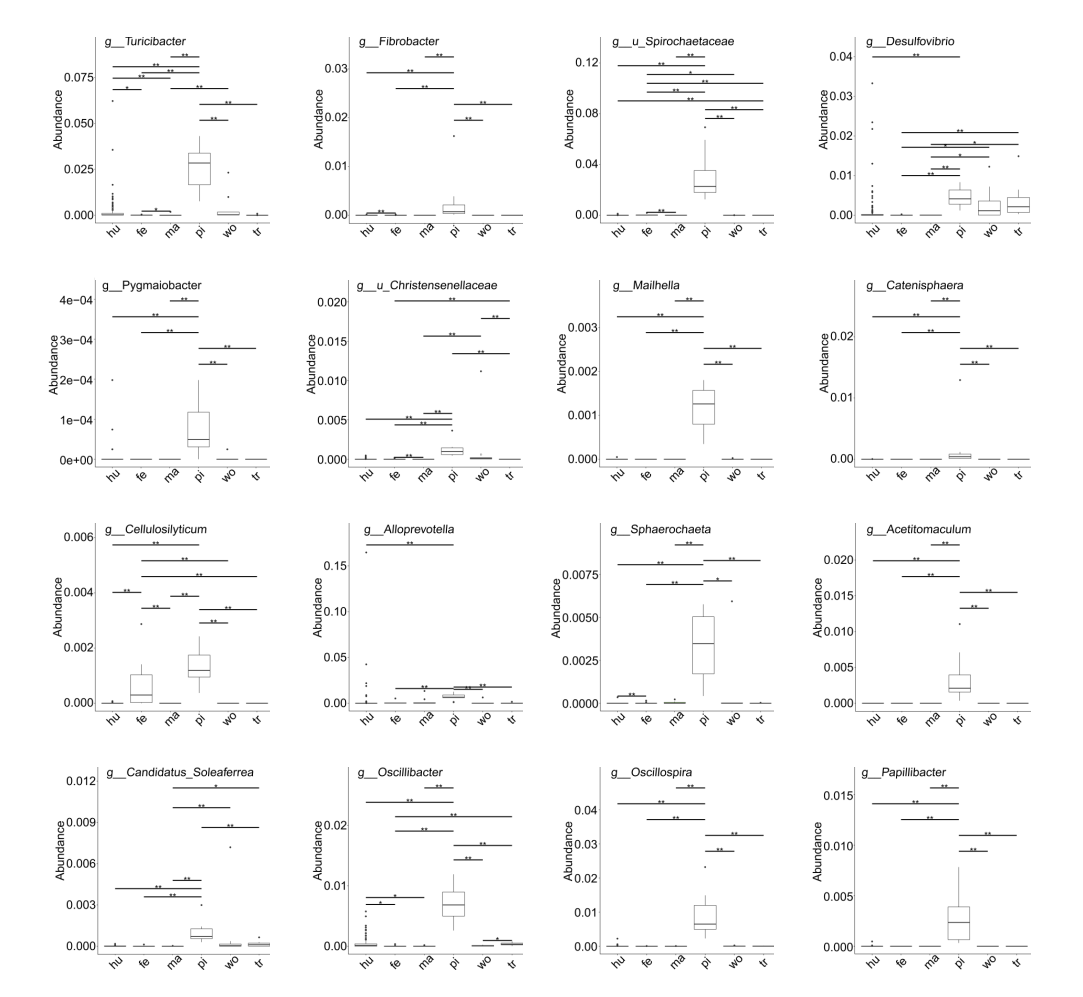


**Fig. S3 Host specific genera detected with Metastat in mini pig** (abundance: frequency in gut microbiota %; ***,** Wilcox p <0.05; ****,** p<0.01)


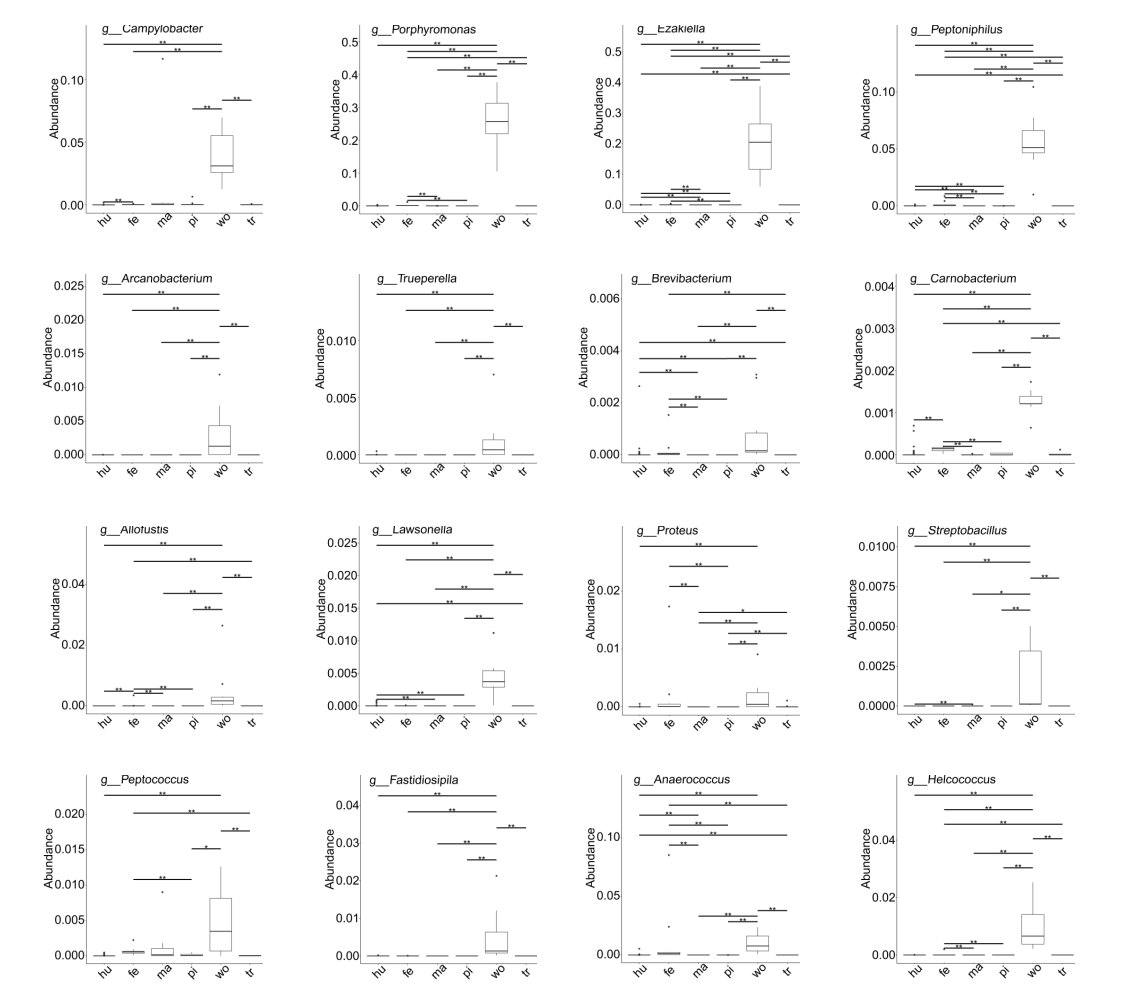


**Fig. S4 Host specific genera detected with Metastat in woodchuck** (abundance: frequency in gut microbiota %; ***,** Wilcox p <0.05; ****,** p<0.01)


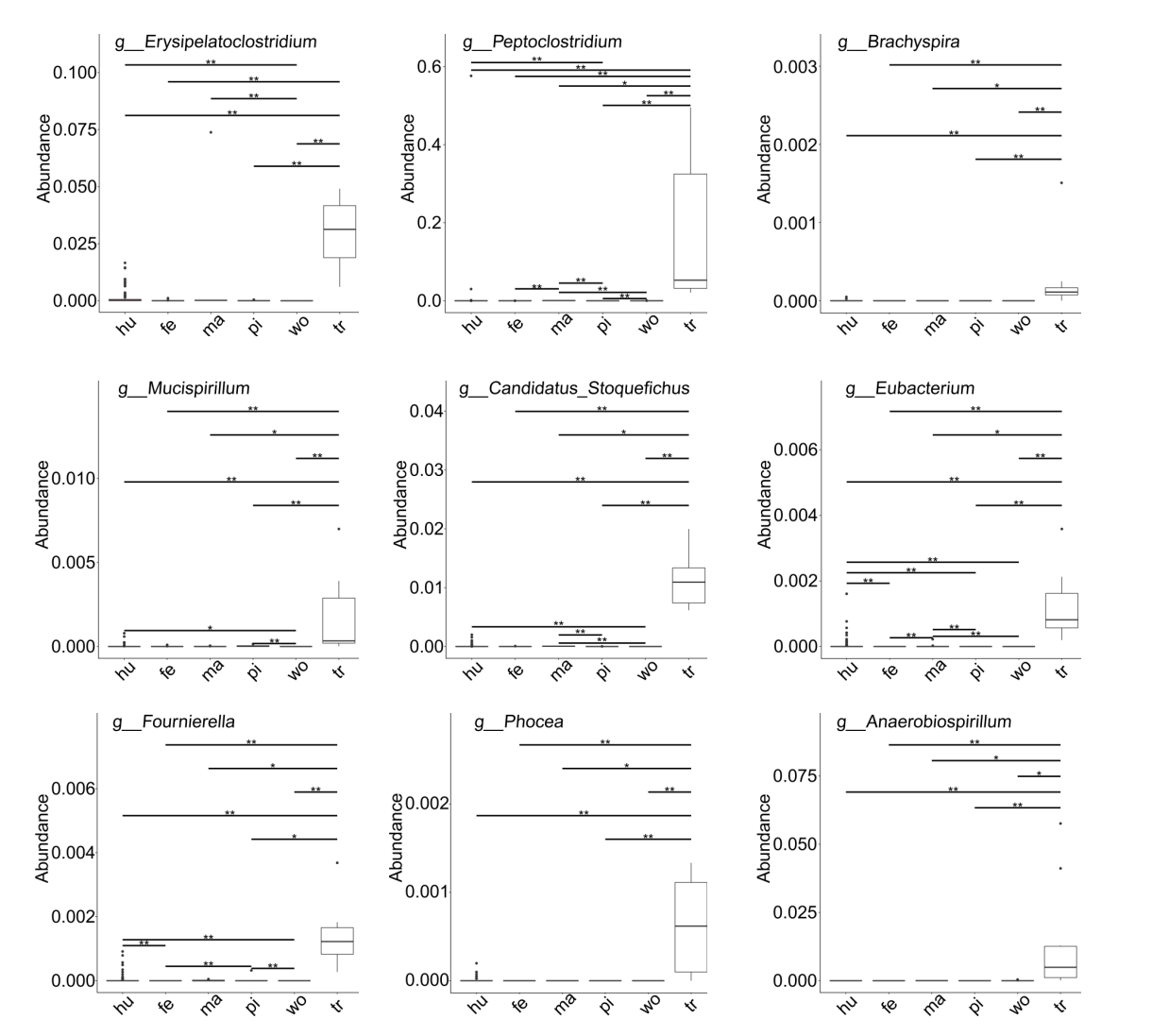


**Fig. S5 Host specific genera detected with Metastat in tree shrew** (abundance: frequency in gut microbiota %; ***,** Wilcox p <0.05; ****,** p<0.01)


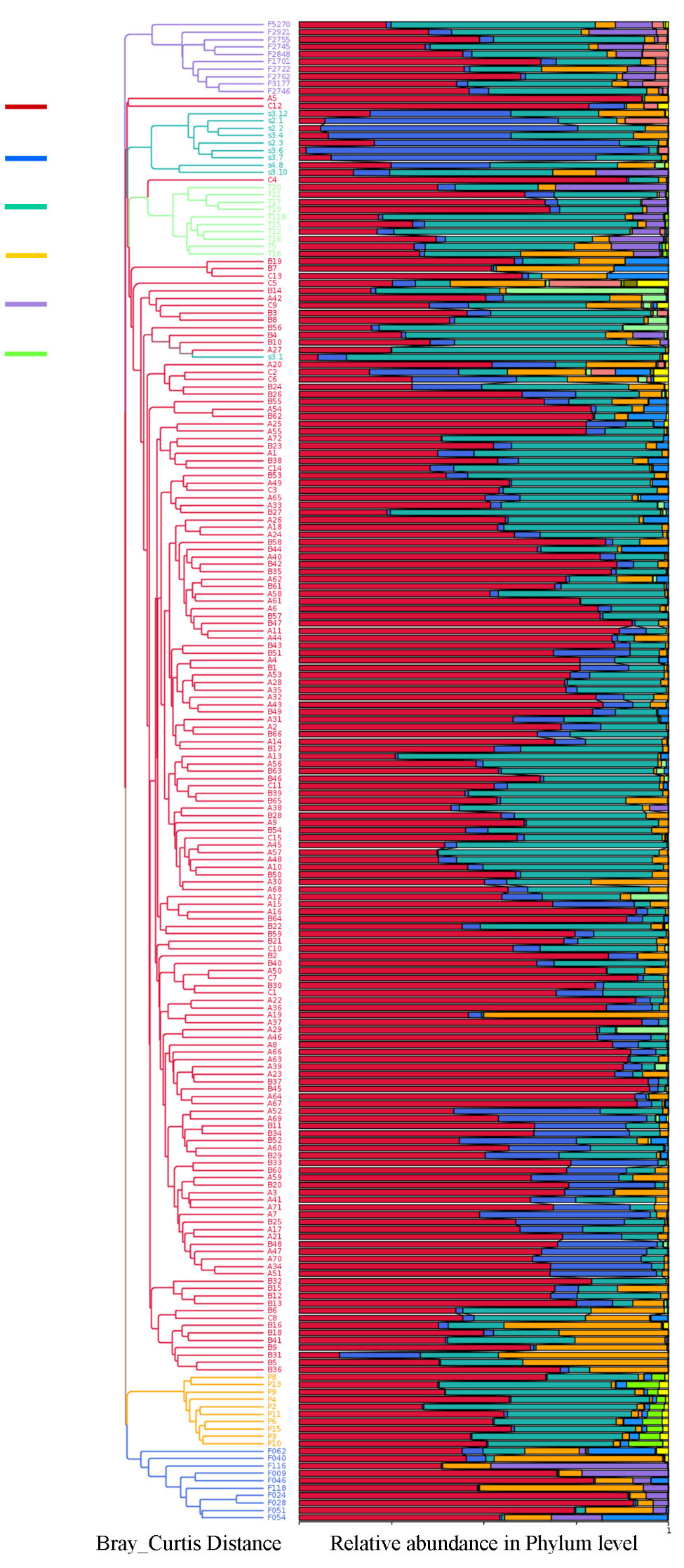


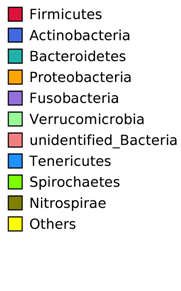


hu

fe

ma

pi

wo

tr

**Fig. S6**

**Hierarchical clustering tree of each sample**.

based on Bray–Curtis dissimilarities.

The samples from different species are distinguished by different line colors. The legend boxes list the 10 most abundant phyla.
